# Food insecurity modifies the association between the gut microbiome and the risk of cognitive impairment in adults

**DOI:** 10.1038/s41514-025-00241-0

**Published:** 2025-06-18

**Authors:** Shoshannah Eggers, Zachary E. Hoggarth, Kiran Nagdeo, Maria J. Banas, Jamil M. Lane, Elza Rechtman, Chris Gennings, Elizabeth O’Neal, Paul E. Peppard, Ajay K. Sethi, Nasia Safdar, Kristen MC Malecki, Amy A. Schultz, Vishal Midya

**Affiliations:** 1 Department of Epidemiology, University of Iowa College of Public Health, Iowa City, IA USA; 2 Department of Environmental Medicine, Icahn School of Medicine at Mount Sinai, New York, NY USA; 3 Department of Epidemiology, New York University School of Global Public Health, New York, NY USA; 4 Department of Community and Behavioral Health, University of Iowa College of Public Health, Iowa City, IA USA; 5 Department of Population Health Science, University of Wisconsin School of Medicine and Public Health, Madison, WI USA; 6 Department of Medicine – Division of Infectious Diseases, University of Wisconsin School of Medicine and Public Health, Madison, WI USA; 7 Department of Environmental and Occupational Health Sciences, University of Illinois Chicago School of Public Health, Chicago, IL USA

**Keywords:** Microbiology, Neuroscience, Social sciences

## Abstract

This study investigated the role of food insecurity as an effect modifier between the gut microbiome, including groups of microbes (cliques), and risk of cognitive impairment (RCI). The analytical sample (*n* = 360) included adult participants of the Survey of the Health of Wisconsin with complete data on food insecurity, RCI, and 16S rRNA sequencing from stool samples. Microbial cliques associated with RCI were identified using an interpretable machine-learning-based algorithm. All analyses were stratified by food insecurity level, and adjusted for relevant confounders. We identified two cliques whose associations with RCI were modified by food insecurity status. The presence of the clique with either *Eisenbergiella* or *Eubacterium* was more strongly associated with RCI for the food-insecure group (β = 0.29, *p* < 0.0001). A clique representing the presence of *Ruminococcus torques*, *Bacteroides*, CAG-352F, and/or *Eubacterium* had a stronger association with RCI for the food-secure group (β = 0.1, *p* < 0.0001). Findings suggest food security be considered in RCI etiology.

## Introduction

Food security, a concept encompassing access, availability, and utilization of nutritionally adequate and safe food, is a global cornerstone of public health. Food insecurity refers to a lack of available financial resources and inadequate access to sufficient food for maintaining an active and healthy lifestyle^1^. In the United States, where abundance often coexists with disparities, issues of food insecurity are persistent, affecting millions across diverse demographics. Over 12% (17.0 million) of U.S. households in 2022 experienced food insecurity at some point throughout the year, showing an increase from the 10.2% (13.5 million) prevalence in 2021^2,3^. Food insecurity is consistently linked to adverse health outcomes such as poorer overall health, increased risk of asthma, limitations in daily activities, particularly among vulnerable populations like children and seniors, heightened risk of chronic diseases like obesity, type 2 diabetes, cardiovascular disease, and adverse neurological health outcomes^4,5^. Food insecurity is inversely associated with cognition and executive function and positively associated with the likelihood of depressive symptoms^6,7^. Analysis of the relationship between poverty, food insecurity, cognitive functioning, and psychological distress revealed that food insecurity significantly mediated the relationship between poverty and both cognitive capacity and psychological distress^8^. Potential mechanisms for this association may include activation of the hypothalamic-pituitary-adrenal (HPA) axis in food-insecure individuals. This HPA activation can release stress hormones that impair memory and executive function by binding to receptors in key brain regions associated with mood and cognition^9,10^.The gut microbiome may also play a role in this association, given its influence on neurological health through the gut-brain axis^11–13^.

Despite significant progress in uncovering the mechanisms linking food insecurity to cognitive health, the relationship among the gut microbiome, food security, and cognition is not yet fully understood. Mohr et. al. investigated the relationship between food security status and the gut microbiome in college students, revealing modest differences in microbial diversity and metabolic pathways between food-insecure and food-secure students^14^. While food insecure students showed microbial metabolism associated with energy harvest, food secure students displayed a higher abundance of genera linked to carbohydrate metabolism. Moreover, low microbial diversity and imbalances between pathogenic (e.g., *Staphylococcus*) and beneficial (e.g., *Bifidobacteria*) microbes may result from calorie-dense, nutrient-poor diets often associated with food insecurity^14–16^. Our previous study found that a higher nutrition score was associated with a wider range of bacterial taxa for those experiencing food insecurity than food security, suggesting that better nutritional quality may have a larger impact on the health of food-insecure individuals via gut microbial mechanisms^17^. These associations may also be transgenerational, as a study following Hurricane Maria in Puerto Rico in 2017 found a decreased abundance of *Veillonella spp*. in infants born to food-insecure mothers compared to those born to food-secure mothers^18^. Together, these findings suggest that food insecurity may alter the composition and metabolism of the gut microbiome, which can produce downstream health consequences. However, not much is known about how groups of multiple microbes, or microbial cliques, are influenced by food insecurity status. While epidemiologic studies of the human microbiome primarily focus on individual taxa or the whole gut microbiome, gut microbes can interact with host health at levels between 1-on-1 and the whole microbiome, e.g. cliques. Previous work from our group has shown that microbial cliques within the gut microbiome are linked to depression and intestinal inflammation, and influenced by environmental exposures^19–21^. Given the links between food insecurity and the gut microbiome, it is likely that food insecurity may influence gut microbial cliques as well.

This study aimed to investigate the role of food insecurity as a modifier between the gut microbiome and the risk of cognitive impairment (RCI) in adults. We hypothesized that food insecurity status influenced the association between the gut microbiome, including gut microbial cliques, and increased RCI. By utilizing data from the Survey of the Health of Wisconsin and its ancillary Wisconsin Microbiome Study and employing Microbiome Co-occurrence Analysis (MiCA), we seek to deepen our understanding of the interplay between food security, the gut microbiome, and cognitive health, with potential implications for public health interventions.

## Results

### Study Population

Within the study sample, 292 (81.1%) did not experience food insecurity, while 68 (18.9%) did (Table 1). The average age among food-secure individuals was 62.6 years compared to an average age of 56.8 years for individuals who are food-insecure. Food-insecure individuals had a greater mean body mass index (BMI) compared to food-secure individuals. Both groups had slightly more females than males. Non-Hispanic White individuals comprised a greater percentage of the food-secure population compared to the food-insecure population. Food-secure individuals were less likely to be pet owners and never smokers. Antibiotic use within the last year was between 30 and 40% for both groups. Cognitive score is higher in the food-secure group than in the food-insecure group.

**Table 1:** Demographic table summarizing the sample under study

	Overall (*n* = 360)	Food-secure (*n* = 292)	Food-insecure (*n* = 68)	*p*-value
**Age mean (sd)**	61.47 (13.44)	62.55 (13.19)	56.84 (13.63)	<0.001
**Race/Ethnicity,** ***n*** **(%)**				
Non-Hispanic White	308 (85.56%)	259 (88.70%)	49 (72.06%)	<0.001
Other	52 (14.44%)	33 (11.30%)	19 (27.94%)	
**Gender,** ***n*** **(%)**				0.89
Female	213 (59.17%)	172 (58.90%)	41 (60.29%)	
Male	147 (40.83%)	120 (41.10%)	27 (39.71%)	
**BMI (kg/m**^**2**^**), mean (sd)**	30.99 (7.37)	30.62 (7.19)	32.58 (8.00)	0.07
**Fiber (g), mean (sd)**	19.06 (10.03)	18.65 (8.86)	20.84 (13.92)	0.57
**Pet owner,** ***n*** **(%)**				0.05
No	172 (47.78%)	132 (45.21%)	40 (58.82%)	
Yes	188 (52.22%)	160 (54.79%)	28 (41.18%)	
**Ever smoker,** **n** **(%)**				0.002
Yes	170 (47.22%)	126 (43.15%)	44 (64.71%)	
No	190 (52.78%)	166 (56.85%)	24 (35.29%)	
**Antibiotic Use in Last Year,** ***n*** **(%)**				0.32
No	236 (65.56%)	195 (66.78%)	41 (60.29%)	
Yes	124 (34.44%)	97 (33.22%)	27 (39.71%)	
**Below 200% of the Poverty Level**				<0.001
Yes	86 (23.89%)	43 (14.73%)	43 (63.24%)	
No	274 (76.11%)	249 (85.27%)	25 (36.76%)	
**Cognitive Score**	2.40 (0.72)	2.42 (0.70)	2.31 (0.82)	0.38
**Shannon alpha diversity**	3.32 (0.45)	3.33 (0.44)	3.26 (0.47)	0.27

### Factors Associated with RCI

**Fig. 1:**
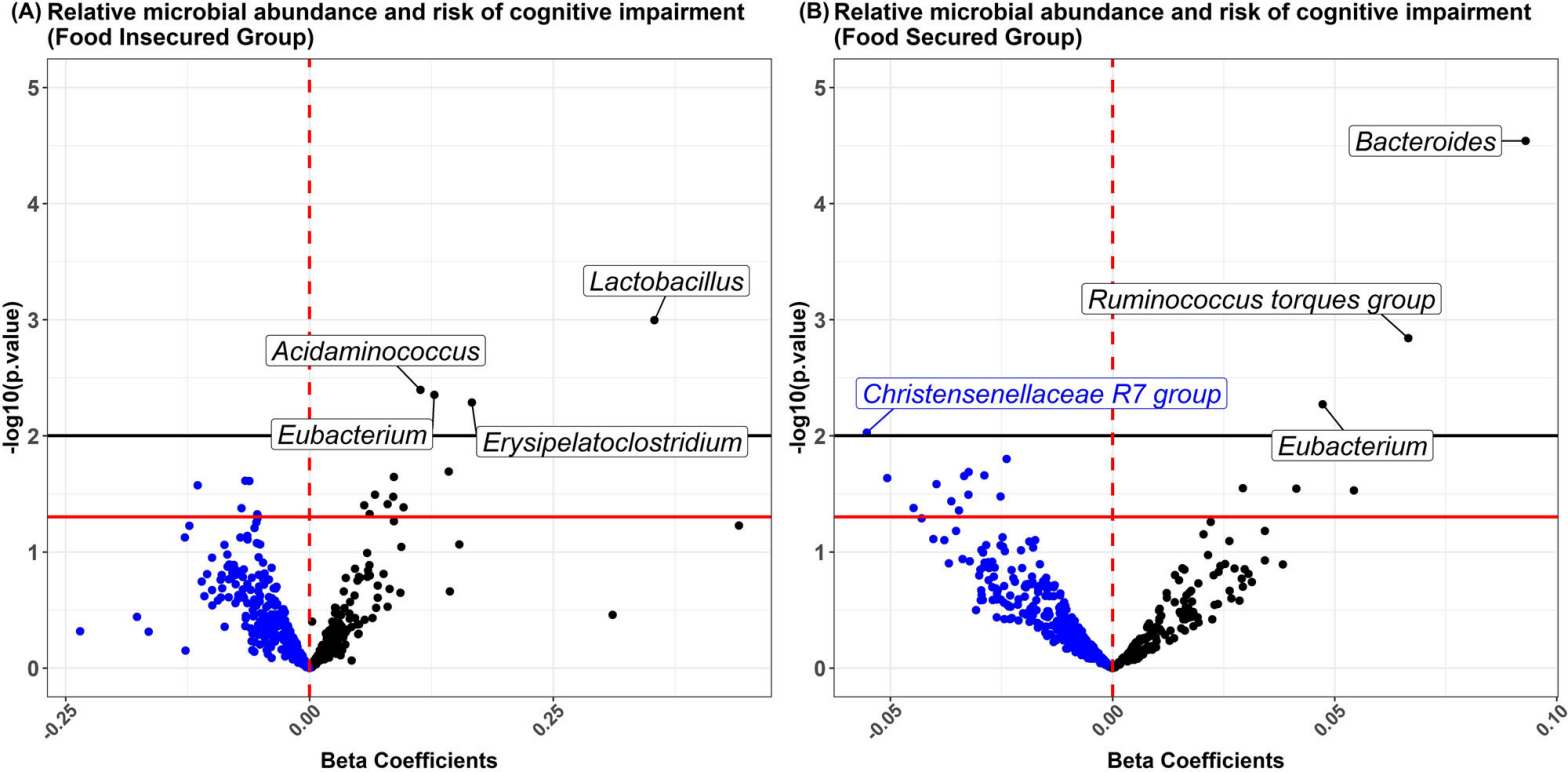
**Associations Between Gut Microbial ASVs and Cognitive Impairment Risk Stratified by Food Security Status.** Volcano plots of association between quartiled relative abundance of gut microbial ASVs (labeled at the genus level) and risk of cognitive impairment in **A** food insecure vs (**B**) food secure group. All regressions were adjusted for covariates. The red and black horizontal lines indicate log-transformed *p*-values at 0.05 and 0.01, respectively.

In covariate-adjusted regression models, food insecurity was associated with an increased RCI (β = 0.06, 95% CI = [0.00, 0.12]). Gut microbiome alpha-diversity had an inverse association with RCI in both the food secure (β = −0.08, 95% CI = [−0.15, −0.02]) and insecure groups (β = −0.09, 95% CI = [−0.26, 0.07]), although the estimate was only statistically significant in the larger food secure group. In adjusted regression models of individual ASVs, a *Bacteroides* ASV was significantly associated with RCI in the food-secure group (β = 0.09, 95% CI = [0.05, 1.36], raw *p*-value < 0.0001), even after the FDR correction. Several other ASVs were associated with RCI (Fig. 1), including a *Christensenellaceae* ASV that was inversely associated in the food secure group; however, they did not maintain statistical significance after correction for multiple comparisons using FDR. The association estimates are presented in Supplementary Table 1.

### Effect Modification by Microbial Cliques

**Fig. 2:**
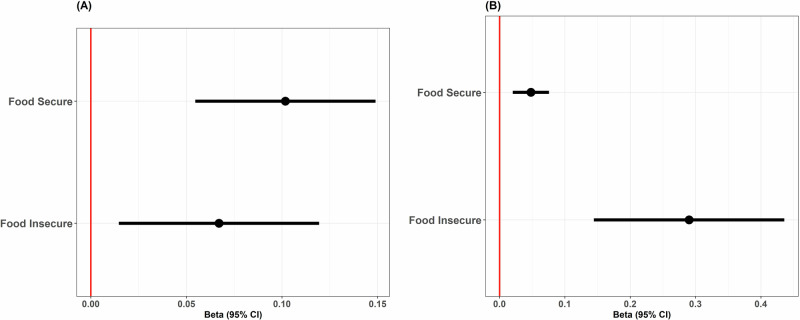
**Cognitive Impairment Risk Associated with Microbial Cliques by Food Security Status.** Adjusted β estimates with 95% confidence intervals for the risk of cognitive impairment in adults with **A** *Ruminococcus torques, Bacteroides, CAG-352F, and/or Eubacterium* clique, and **B** *Eisenbergiella* and/or *Eubacterium* clique stratified by food security. The red horizontal line denotes a null value of the β estimate.

Following MiCA analyses, two different microbial cliques were identified as associated with risk for cognitive decline. The list of microbial co-occurrences is presented in Supplementary Table 2. The first clique consisted of *Eisenbergiella* and/or *Eubacterium*. Of 68 food insecure individuals, 17 (25%) harbored this clique, the same proportion within the food secure group, 73/292 (25%). A second clique was identified consisting of *Ruminococcus torques, Bacteroides, CAG-352F*, and/or *Eubacterium*. This clique was found in 23/68 (33.8%) of food-insecure individuals, compared to 98/292 (33.6%) in those who are food-secure. Among food-insecure individuals, harboring a clique of *Eisenbergiella* and/or *Eubacterium*, was significantly associated with increased RCI (β = 0.29, 95% CI = [0.14, 0.44], robust *p* < 0.0001), as depicted in Fig. 2. There was a significant but much weaker association with RCI observed with this same clique for people food-secure individuals (β = 0.05, 95% CI = [0.02, 0.08], robust *p* < 0.001). Individuals experiencing food insecurity who had a clique consisting of *R. torques, Bacteroides, CAG-352F*, and/or *Eubacterium* were significantly associated with increased RCI (β = 0.07, 95% CI = [0.01, 0.12], robust *p* = 0.01). This association was slightly stronger among individuals that were food-secure (β = 0.10, 95% CI = [0.05, 0.15], robust *p* < 0.0001). These results are summarized in Supplementary Table 3. Further, we tested the interaction terms (microbial clique by food insecurity status) directly in a large single overall regression model. The results are presented in the Supplementary Table 4. The directionality of the interaction terms and strength of association lend further evidence toward the microbial cliques and food insecurity interaction.

## Discussion

To our knowledge, this is the first epidemiologic study to assess the modifying role that food insecurity may play on the relationship between the gut microbiome and RCI. Our findings showed that for food-insecure individuals, harboring a clique of *Eisenbergiella* and/or *Eubacterium* was significantly associated with RCI; however, the association was weaker in the food-secure group. Alternatively, for food-secure individuals, having a clique of *R. torques, Bacteroides, CAG-352F*, and/or *Eubacterium* was significantly associated with RCI. In contrast, the association was weaker in the food-insecure group. This finding may be particularly important while devising microbial interventions for cognitive impairment, as food security status may alter the effectiveness of such interventions.

There are many possible mechanisms by which food insecurity could modify the effect of the gut microbiome and cognitive impairment, especially considering that food insecurity may be a long term exposure, exerting health effects across the lifespan. Compositional and metabolic shifts in the gut microbiome associated with food insecurity may lean towards energy harvest rather than neurotransmitter and neuromodulator production. For instance, reduced production of short-chain fatty acids (SCFAs), commonly neuromodulators produced by the gut microbiome that also promote epithelial barrier integrity, leads to increased permeability of the gut, resulting in bacterial metabolites entering the circulatory system and triggering an immune response^22,23^. It is hypothesized that bacterial metabolites such as endotoxin and lipopolysaccharide pass through the gut and blood-brain barriers and contribute to neuroinflammation and degradation, potential precursors for dementia and cognitive decline^24^. Additionally, environmental stressors such as pollution, hazardous occupational exposures, and lack of green space are all associated with food insecurity and can modulate the gut microbiome and its functionality^25–28^.

Several of the bacteria we identified in these cliques are known to cross-feed, meaning they either breakdown complex molecules that other bacteria metabolize, or they rely on metabolites produced by other bacteria in the gut microbiome. For instance, *R. torques*, considered a keystone species in the gut microbiome, is a mucin degrader, producing monosaccharides that are used by other bacteria^29^. Likewise, Bacteroides species are known to be able to utilize many different available nutrients from simple sugars to complex polysaccharides^30^. They can play the producer role by breaking down large molecules, as well as the end user role by metabolizing simple sugars. Eubacterium species are also known to cross-feed, metabolizing carbohydrate intermediates like acetate and lactate, produced by other bacteria, into butyrate and propionate^31^. These well documented cross-feeding relationships support the biological plausibility of the cliques identified in our study.

Previous studies have found relationships between shifts in the microbiome and adverse neurocognitive outcomes, including dementia^32,33^. Bacteria in the genera *Eubacterium* and *Eisenbergiella* have been associated with neurodegenerative diseases, both as risk and protective factors. A small study of gut microbes in dementia patients found that *Eubacterium hallii* was associated with dementia; however, the abundance of *Eubacterium rectale* was lower in dementia patients compared to controls^34^. Furthermore, that same study found that increasing abundance of the genus *Eisenbergiella* was associated with increased severity of cognitive impairment^34^. Conversely, in a large case-control study of Alzheimer’s Disease (AD), multiple species of *Eubacteria* were protective against AD, and *Eisenbergiella* was identified as the single strongest protective factor against AD in a meta-analysis of two case-control studies^35^. In a case-control study of mild cognitive impairment (MCI), *Eisenbergiella* was found to be protective against MCI for men and a risk factor for MCI for women^36^. Considering our findings that food security may alter the association between these bacterial cliques and RCI, a more nuanced precision environmental health approach may be needed for effective microbial interventions for cognitive health.

Recent microbiome studies have also found associations between *R. torques* and cognitive impairment. One study determined that the relative abundance of *R. torques* was increased in dementia patients with Lewy body, a progressive dementia resulting in declining cognitive function^37^. In a smaller case-control study of cognitive impairment, *R. torques* was found to be a predictive marker of cognitive impairment^38^. Another study of MCI in Parkinson’s Disease patients found the *Runimococcus* genus, including species besides *R. torques*, were more abundant in controls than those with MCI. *Bacteroides* have also been linked to cognitive outcomes, with several studies identifying Bacteroides in association with RCI and age-related cognitive decline^39,40^. A meta-analysis of AD studies found regional variations in the association between *Bacteroides* and AD, with associations found in the US cohorts but not in the Chinese cohorts^41^. It was hypothesized that these regional differences occur due to differences in dietary patterns and access to food, possibly supporting our findings.

There are some limitations that should be considered when interpreting the findings of this study. While an investigation of more than 300 individuals is large for a microbiome study, there is a potential lack of power for detecting weak associations due to sample size, particularly in stratified analyses. Additionally, all study participants were sampled from Wisconsin, which may limit the generalizability of the findings to other places with different demographics, environmental considerations, and dietary patterns. There is also a potential for misclassification of RCI when considering external factors such as stress, amount of sleep, or mental health problems that could have played a role in how an individual scored on the adapted mini-cog^42^. Another limitation is potential unmeasured confounding variables, such as environmental or occupational factors not captured or adjusted for in our analyses. Finally, due to this study being cross-sectional in nature, there is no temporality in the investigation, hindering our ability to infer causation between gut microbial cliques and RCI.

This study has highlighted the importance of considering food insecurity in developing prevention strategies against cognitive impairment. A strength of this study is considering the interplay of multiple domains – gut microbiome, food insecurity, and cognitive impairment. Further, the use of MiCA, in addition to the analysis of alpha diversity and individual taxa, adds to the robustness of the result^19^. MiCA allowed us to identify associations between groups of bacteria and RCI, potentially indicating combined effects. However, further investigation may focus on mechanisms by which microbial cliques affect the central nervous system and how food insecurity modifies those pathways. These endeavors can help explain the complex relationship between food insecurity, the gut microbiome, and neurocognitive outcomes, leading to more effective public health interventions to prevent cognitive impairment.

## Methods

### Data Source and Study Population

The Survey of the Health of Wisconsin (SHOW), which collected data from 2008 to 2023, is a statewide health examination survey conducted in Wisconsin^43,44^. SHOW aimed to gather data on health exposures, outcomes, and various determinants of health, such as healthcare access, social factors, lifestyle choices, and behavioral patterns. SHOW collected survey data and objectively measured body characteristics, as well as biological specimens. In 2016, the SHOW protocol was expanded to include the Wisconsin Microbiome Study (WMS)^45^, which recruited participants aged 18 and older and collected samples, including stool, for microbiome analysis. The data used in this analysis were sourced from survey responses and stool samples collected in 2016 and 2017. The protocols for SHOW and WMS received approval from the University of Wisconsin Institutional Review Board, and all participants provided written consent to participate.

### Survey Data

Food insecurity was assessed using three questions: (1) In the last 12 months, did you ever get emergency food from a church, a food pantry, or a food bank, or eat in a soup kitchen? (2) In the last 12 months, have you been concerned about having enough food for you or your family? (3) In the last 12 months, were you authorized to receive Food Stamps, which include a food stamp card, voucher, or cash grants from the state for food? If participants responded with “yes” to any of these questions, they were considered to be food insecure, and all other participants were considered food secure^17,43^.

SHOW participants completed an adapted “mini-cog” test to assess RCI^42^. Participants were given a word-recall test, which included memorizing three words and recalling these words after completing a drawing of a clock set to a specific time. Results ranged from a count of 0 to 3 based on the number of correctly remembered words. Increasing scores represent a lower likelihood of RCI^42,46^. However, in regression models, the cognitive test scores were inverted and then log-transformed to account for skewness for ease of interpretation. We use these inverted and log-transformed cognitive test scores as our outcome (and therefore, a higher value indicates increasing RCI).

Potential confounders included in our analyses were age, and BMI, based on measured height and weight, and dietary fiber, all treated continuously. Dietary fiber consumption in grams was calculated based on the National Cancer Institute’s Diet History Questionnaire II^47^. Categorical variables included self-reported gender (male/female), race/ethnicity (non-Hispanic White/other), pet ownership (yes/no), smoking history (yes/no), and use of antibiotics in the past year (yes/no).

### Gut Microbiome

Details of the microbiome sample collection, analysis, as well as data processing, have been reported previously^45,48,49^. Participants were responsible for self-collecting a stool sample at home using a kit provided by SHOW. Samples collected were shipped to and received at the Infectious Disease Research Laboratory located at the University of Wisconsin—Madison within a 24-hour timeframe, where they were processed and frozen at -80°C for future use. Genomic DNA was extracted using a combination of chemical, heat, and mechanical lysis methods, followed by purification using a phenol-chloroform-isoamyl alcohol extraction and clean-up kit. DNA quantity was assessed before PCR amplification of the 16 s rRNA V4 region using custom barcoded PCR primers^28^. Amplicon sequences were purified through agarose gel electrophoresis and a 96 well cleanup kit, quantified, and pooled for sequencing on an Illumina MiSeq platform according to the manufacturer’s instructions. Sequencing data was processed using QIIME 2 v2021.4^50^. Sequences were imported using Casava 1.8 and denoised with DADA2 v.1.18.0^51^, which generated amplicon sequence variants (ASVs). ASVs were assigned taxonomy from the Silva_138 database^52^ using the classify-sklearn naïve Bayes taxonomy classifier v0.24.1^53^. Contaminants from the negative controls were removed using Decontam^54^, and Eukaryotic, chloroplast, mitochondrial, and unassigned ASVs were also removed. Samples were subsampled to an even depth of 8396 after removing any samples with fewer than 5000 sequencing reads. Shannon diversity^55^ was calculated using the Vegan package^56^.

### Statistical Analysis

All statistical analyses were conducted in R (version 4.2.3). Microbiome abundance values were converted to relative abundance per individual. To ensure robustness, only bacterial taxa with more than 5% prevalence were included in the analysis. Relative abundances were not rescaled after the removal of rare taxa. The analytical sample included 360 participants who had complete data on all variables. Descriptive statistics were calculated for all analytical variables using the Wilcoxon rank sum test or Fisher’s exact test, where appropriate. We first conducted some preliminary analyses using simple linear regression (1) to estimate the association between food insecurity and RCI and (2) the association between Shannon alpha diversity and RCI. Further, we conducted multiple separate regressions for each ASV in the microbiome in association with RCI for the food-secure and insecure groups. The results were then presented using volcano plots. The raw unadjusted *p*-values were corrected for the false discovery rate. Each ASV was converted to quartiles so that association estimates were comparable in the volcano plots. All analyses were adjusted for age, race/ethnicity, BMI, gender, pet ownership, smoking, amount of fiber consumed, and antibiotic use.

Our main analysis included the MiCA analysis, an interpretable machine-learning framework to identify co-occurring bacterial taxa or microbial cliques associated with RCI. Separate stratified analyses were conducted for the food-secure and food-insecure groups. MiCA was conducted in two stages^19^, first, we used a machine learning algorithm to discover microbial cliques predictive of higher RCI. Using a regression-based framework, in the second stage, we estimated the associations between the joint relative abundance of the identified microbial cliques and RCI. In the first stage, we used the repeated holdout signed-iterative Random Forest (rh-SiRF) algorithm^19,57^ with ASV abundance as the predictor and these inverted and log-transformed cognitive test scores as our outcome (higher values indicate higher risk). This tree-based method identifies the most frequently occurring combinations throughout the generated forest by parsing through each branch. Therefore, any predictive non-linear and non-additive combination of microbial cliques can be identified through this algorithm. Next, to ensure the identified cliques are not overfitted, we repeated the algorithm 1000 times, with 250 bootstraps on a 60% training and 40% test data partition. Bootstrapping and repeating the algorithm a large number of times ensures that the true microbial clique combination repeatedly occurs multiple times. In contrast, if there is no possible strong signal, no microbial clique combination will occur a substantial number of times. Based on the highest frequency of occurrences of the combinations across repeated holdouts and bootstraps, we identified the top combinations of co-occurring bacteria so that the combinations form a closed-loop network^20,21^.

This analysis was stratified by food-insecurity status; therefore, we obtained two combinations of co-occurring bacteria and created a joint indicator variable that sums up the presence of each microbe within each of the identified cliques. For interpretability and to include as many samples as possible, we constructed a clique indicator variable that sums up the number of identified clique ASVs present for each participant. Thus, for a clique of two ASVs, values range from 0–2, and for a clique of four ASVs, values range from 0–4, indicating the presence of any ASV within the clique. These clique variables are then used as the primary exposure variable in covariate-adjusted regression models of RCI. Note that since this analysis was stratified by food-insecurity status, the identified microbial clique in one group was then validated using a regression in the out-of-bag sample of the other group, which in turn increases the robustness of our results.

The outcome was inverted for interpretability and log-transformed for rapid convergence for the large sample properties to kick in. We estimated all the main *p*-values for microbial cliques produced by the linear regressions by permuting the outcome 10^5^ times for each regression to eliminate reliance on large-sample asymptotic assumptions of normality^19^. Any two-tailed *p*-value less than 0.05 was considered statistically significant. Only samples with complete data on food insecurity status and cognitive score were added to the analysis. Less than 2% of a few covariates were missing, which had been imputed using the predicted mean matching as implemented in the “mice” R package^58,59^. As a sensitivity analysis, we conducted rigorous covariate balancing using a sub-class matching technique and love plots on each clique-based regression analysis to ensure minimal influence of bias due to confounding^60^.

## Supplementary information

Supplementary Material

## Data Availability

All data generated and analyzed from this study came from the Survey of the Health of Wisconsin and its ancillary Wisconsin Microbiome Study. All sequences associated with this study have been deposited into the National Center for Biotechnological Information’s Short Read Archive and are available under BioProject ID PRJNA999362. Epidemiologic data is available by request through https://reach.med.wisc.edu/.
