# Supplementary material for: Food insecurity modifies the association between the gut microbiome and the risk of cognitive impairment in adults
Source: NPJ Aging. 2025 Jun 18;11(1):47. doi: 10.1038/s41514-025-00241-0 (PMC12177064; doi:10.1038/s41514-025-00241-0)
Supplement: Supplementary file 1 — Supplementary Material [file 41514_2025_241_MOESM1_ESM.pdf]

Food Insecurity Modifies the Association Between the Gut Microbiome and the Risk of Cognitive Impairment in Adults

Shoshannah Eggers, PhD,[1] Zachary E. Hoggarth, MPH,[1] Kiran Nagdeo, MPH,[2,3] Maria J. Banas, MS,[1] Jamil M. Lane, PhD,[2] Elza Rechtman, PhD,[2] Chris Gennings, PhD,[2] Elizabeth O'Neal, PhD,[4] Paul E. Peppard, PhD,[5] Ajay K Sethi, PhD,[5] Nasia Safdar, MD, PhD,[6] Kristen MC Malecki, PhD,[7] Amy A. Schultz, PhD,[5] Vishal Midya, PhD, MSTAT[2]

1. Department of Epidemiology, University of Iowa College of Public Health, Iowa City, IA, USA

2. Department of Environmental Medicine, Icahn School of Medicine at Mount Sinai, New York, NY, USA

3. Department of Epidemiology, New York University School of Global Public Health, New York, NY, USA

4. Department of Community and Behavioral Health, University of Iowa College of Public Health, Iowa City, IA, USA

5. Department of Population Health Science, University of Wisconsin School of Medicine and Public Health, Madison, WI, USA

6. Department of Medicine – Division of Infectious Diseases, University of Wisconsin School of Medicine and Public Health, Madison, WI, USA

7. Department of Environmental and Occupational Health Sciences, University of Illinois Chicago School of Public Health, Chicago, IL, USA

Corresponding Author: Shoshannah Eggers, shoshannah-eggers@uiowa.edu

Supplementary Table 1: Association estimates of quartiled relative abundance of gut microbial ASVs (labeled at the genus level) and risk of cognitive impairment in (A) food insecure vs (B) food secure group (from Figure 1)

Group	Genus	Family	β	SE	t-value	p-value	FDR
Food Secured Group	Bacteroides	Bacteroidaceae	0.09	0.02	4.25	$2.88X10^{-5}$	0.01
	Ruminococcus torques group	Lachnospiraceae	0.07	0.02	3.22	$1.44X10^{-3}$	0.32
	Eubacterium	Eubacteriaceae	0.05	0.02	2.81	$5.36X10^{-3}$	0.79
	Christensenellaceae R7 group	Christensenellaceae	-0.06	0.02	-2.62	$9.36X10^{-3}$	0.91
Food Insecure Group	Lactobacillus	Lactobacillaceae	0.35	0.10	3.46	0.001	0.45
	Acidaminococcus	Acidaminococcaceae	0.11	0.04	2.99	0.004	0.57
	Eubacterium	Eubacteriaceae	0.13	0.04	2.96	0.004	0.57
	Erysipelatoclostridium	Erysipelatoclostridiaceae	0.17	0.06	2.90	0.005	0.57

The estimates of Beta, SE, and t-value are rounded up to two digits after the decimal point. The results are presented for all the associations with unadjusted p-values below 0.01; FDR: False discovery rate

Supplementary Table 2: Top 10 microbial co-occurrences from the MiCA algorithm

Group	Microbial co-occurrences	%of co-occurrences
Food Secured Group	*Bacteroides- Ruminococcus torques group*	43
	Bacteroides - CAG-352F	37
	Eubacterium - Ruminococcus torques group	26
	Bacteroides - Eubacterium	23
	CAG-352F - Ruminococcus torques group	23
	Bacteroides – Eubacterium nodatum_group	21
	Bacteroides - Lachnospiraceae	20
	Lachnospiraceae - Ruminococcus torques group	20
	CAG-352F - Eubacterium	17
	Eubacterium nodatum_group – Ruminococcus torques group	17
Food inecure Group	*Eubacterium - Eisenbergiella*	21
	Bacteroides- Eisenbergiella	20
	Phascolarctobacterium- Eisenbergiella	16
	Agathobacter- Eisenbergiella	15
	Eisenbergiella- Lachnospiraceae	15
	Eisenbergiella- Streptococcus	14
	Eisenbergiella- Catenibacterium	14
	Agathobacter- Eisenbergiella	13
	UCG-002 - Eisenbergiella	8
	Eubacterium – Bacteroides	8

The food-secured group had 769 co-occurring microbial combinations, whereas the food-insecure group had 267 co-occurring microbial combinations. For the food-secured group, we also identified three ordered microbial co-occurrences: (1) *Bacteroides- CAG-352F- Eubacterium* (3% co-occurrence), and (2) *Bacteroides- CAG-352F- Ruminococcus torques group* (3% co-occurrence. All the downstream two-ordered microbial co-occurrences of the four-ordered co-occurrence are within the top 10, and there is evidence of further three-ordered co-occurrence (downstream from the same four order), as noted here. For the food-insecure group, *Eubacterium - Eisenbergiella* forms a closed group. Although Eubacterium – Eisenbergiella – Bacteroides can be used as a closed-loop microbial clique, the multiple occurrences of *Eisenbergiella* throughout raises the chances of false positives. Therefore, we made a conservative choice and chose only *Eubacterium – Eisenbergiella* as the closed loop clique. The two-ordered microbial co-occurrences that form the closed loop are italicized.

Supplementary Table 3: Association estimates from *Figure 2*

Group	Genus	β	SE	95% CI	Permutation-based robust p-value
Clique of Ruminococcus torques, Bacteroides, CAG-352F, and/or Eubacterium	***Food Secured group***	0.10	0.02	0.05, 0.15	3X 10^{-5}
	Food Insecure Group	0.07	0.03	0.01, 0.12	0.01
Clique of Eisenbergiella and/or Eubacterium	***Food Secured group***	0.05	0.01	0.02, 0.08	7X 10^{-4}
	Food Insecure Group	0.29	0.07	0.14, 0.44	< 10^{-5}

The permutation-based p-value was repeated 10^5 times

Supplementary Table 4: Association estimates from microbial clique and food security status interaction terms from the overall model

Interaction terms	β	SE	95% CI	Model-based p-value	Permutation-based robust p-value
Food insecurity indicator: Clique of Ruminococcus torques, Bacteroides, CAG-352F, and/or Eubacterium	-0.15	0.08	-0.31, 0.02	0.08	<10^{-4}
Food insecurity indicator: Clique of Eisenbergiella and/or Eubacterium	0.23	0.10	0.03, 0.44	0.03	<10^{-4}

The permutation-based p-value was repeated 10^4 times
